# Micromanaging Glucose Tolerance and Diabetes

**DOI:** 10.15171/apb.2017.066

**Published:** 2017-12-31

**Authors:** Negar Saliani, Soheila Montazersaheb, Shideh Montasser Kouhsari

**Affiliations:** ^1^Department of Cellular and Molecular Biology, School of Biology, College of Sciences, University of Tehran, Tehran, Iran.; ^2^Stem Cell Research Center, Tabriz University of Medical Sciences, Tabriz, Iran.

**Keywords:** β-cell function, Glucose homeostasis, MicroRNA, MicroRNA biomarkers

## Abstract

MicroRNAs (miRNAs) are endogenous non-coding RNAs that have significant roles in biological processes such as glucose homoeostasis. MiRNAs fine-tune target genes expression via sequence-specific binding of their seed sequence to the untranslated region of mRNAs and degrade target mRNAs. MicroRNAs in islet β-cells regulate β-cell differentiation, proliferation, insulin transcription and glucose-stimulated insulin secretion. Furthermore, miRNAs play key roles in the regulation of glucose and lipid metabolisms and modify insulin sensitivity by controlling metabolic functions in main target organs of insulin such as skeletal muscle, liver and adipose tissue. Moreover, since circulating miRNAs are detectable and stable in serum, levels of certain miRNAs seem to be novel biomarkers for prediction of diabetes mellitus. In this article, due to the prominent impact of miRNAs on diabetes, we overviewed the microRNAs regulatory functions in organs related to insulin resistance and diabetes and shed light on their potential as diagnostic and therapeutic markers for diabetes.

## Introduction

MicroRNAs (miRNAs) are one of epigenetic mechanisms that modulate different biological processes‏ via silencing‏ and in some cases activating gene expression.^1,2^ miRNAs are 18–25 nucleotides long and these non-coding RNA molecules are involved in post-transcriptional regulation of large number of genes in various organisms (up to 30% genes). This class of RNAs has highly conserved structure. The first miRNA, lin-4, was discovered in nematode *Caenorhabditis elegans,* less than 40 years ago.^3,4^ After then, different groups have found miRNAs in some plants and metazoa.^5,6^

MiRNAs degrade target mRNA or inhibit protein translation in order to inactivate their target genes. This function occurs by binding the “seed sequence” 2–8 nucleotides at 5′ end of miRNA to “untranslated sites” the 3 ′ UTR of the target mRNA. However 5 ′ UTR, promoter elements or coding sequences of target genes are interaction regions for seed sequences.^6^ Depending on the binding quality, the mechanism of regulation is different. In the perfect binding, RNA-induced silencing complex (RISC) is active and fragmentizes the target mRNA, but in weak binding situation miRNA interferes the ribosome assembly or leads to early detachment of ribosome from mRNA. Also, exonucleolytic digestion can happen via deadenylation and decapping of target mRNA.^7,8^

Several miRNAs can recognize a distinct gene and interact with it. On the other hand, a single miRNA can bind lots of genes. There is not a same annotation criteria to analyze miRNAs, so their number is not exactly clear. Actually, about 2500 well known miRNAs have been found in the human genome.^9^ Each miRNA is assigned a name‏ and registered to miRNAs catalog which is available in miRBase database (www.mir base.org; v21,June2014).^10^

## MicroRNA biogenesis

Three major enzymes are involved in miRNA biogenesis, RNA polymerase II, ribonuclease III enzymes (RNase -III), Drosha and Dicer, which act in nucleus and cytoplasm respectively. At the first step, RNA polymerase II transcribes miRNA and produces primary-miRNA (pri-miRNA) which contains a stem-loop structure where the dsRNA-binding protein named DGCR8 in humans, and Pasha in *Drosophila melanogaster* and C*aenorhabditis elegans* and Drosha (micro-processor complex), cleave it into miRNAs (pre-miRNA).^9,11,12^ This cleavage is done in both strands of the stem near the base of the primary stem-loop. Some regulatory proteins such as SMAD (small mothers against decapentaplegic) proteins, the signal transducers of TGFB/ BMP (transforming growth factor beta/ bone morphogenetic protein) accompany RNase III endonuclease to regulate its function.^13^

At the second step, cytoplasmic processing begins by RNase III endonuclease, Dicer1. XPO5 is responsible for transfer of pre-miRNAs from nucleus to cytoplasm. Cofactor of XPO5 is Ran-guanosine triphosphate– dependent nucleo/cytoplasmic cargo.^14^ Dicer1 in presence of dsRNA binding protein, TARBP2 [TAR (HIV-1) RNA binding protein 2], produces a small double stranded miRNA by digesting the loop side of the pre-miRNA hairpin.^15^

MiRNA maturation is accomplished by helicases which unwound this truncated double strand miRNA to produce a single strand. The single strand RNA is active and enters Argonaute (AGO) to destroy target mRNAs. Generally, “guide” strand or “-3p” is functional and “passenger” strand, or star strand or “-5-p” is destroyed.^16^ A large number of mature miRNAs act in cytoplasm after maturation but some of them have capacity to control the gene expression in other cells. They can release into biological fluid or cell culture media.^17^

## MicroRNAs acting on pancreatic β-cells differentiation and functions

The differentiation of insulin-producing β-cells, the highly proficient cells, is orchestrated with several signaling pathways and molecular mechanisms from fetal period until weaning. The ample evidence declares that miRNAs play pivotal roles in β-cell differentiation and functions. Importantly, generation of Dicer-null β-cells resulted in a complete loss of insulin-secreting cells.^18,19^ β-cell specific deletion of Dicer1 using the rat insulin promoter 2 (RIP)-Cre transgene led to impaired pancreas development, declined β-cell mass and insulin secretion.^20^ In line with this evidence, recent study on βDicer-null mice indicated that miRNA loss primarily afflicts β-cell secretory function prior to any decrease in insulin content or β-cell mass.^21^

Investigations for finding the exhaustive list of miRNAs in β-cell differentiation are presently in the limelight. In 2004, the pioneering study demonstrated that miR-375 is highly expressed in pancreatic β-cells and contributes to the β-cell differentiation, pancreas development, insulin biogenesis, insulin secretion and generates the β-cell identity.^22^ Over-expression of miR-375 induces human embryonic stem cells differentiation into islet β-cells in culture in a stepwise process and its expression pattern resembles that of the human fetal pancreas.^23^

Furthermore, up-regulation of miR-375 following treatment with anti-miR-9 in human bone marrow mesenchymal stem cells induces differentiation into mature islet like clusters and improves insulin secretion in a glucose-regulated manner by virtue of controlling the levels of key transcription factors involved in pancreatic β-cells maturation such as SOX-17 and HNF-3 beta/FoxA2.^24^ Nathan et al. showed that expansion of human islet β-cells from adult human pancreatic islets is usually skewed due to the modifications in miRNAs expression. They demonstrated that over-expression of miR-375 in β-cell-derived cells redifferentiated them to cells with more β-cell functional phenotype.^25^ Also, it was found that miR-375 and miR-184 form a network with AGO2 to regulate β-cell expansion.^26^

Emerging data suggests that miR-124a, plays a prominent role in β-cell differentiation and insulin secretion. Expression of miR-124a elevates during mouse embryonic pancreas development. FoxA2, a transcription factor essential for β-cell differentiation and insulin secretion, is a target of miR-124a.^27^ Consistent with this finding, Neurog3, a major β-cell transcription factor which regulates pancreas development and differentiation, was identified as a specific target of miR-124a.^28^

Glucose-stimulated insulin secretion (GSIS) is a critical process which controls the metabolic homeostasis. Several miRNAs are directly involved in GSIS.^29^ MiR-375 inhibits GSIS at the late stages of exocytosis in pancreatic β-cells by targeting myotrophin.^22^ cAMP as a second messenger, increases insulin secretion in the presence of glucose and activates PKA, which subsequently phosphorylates and activates downstream targets in order to enhance GSIS.^30,31^ It has been shown that reduced miR-375 expression enhances GSIS via cAMP/PKA-dependent or PKA-independent pathway.^32,33^

Mir-9 negatively regulates GSIS by targeting Sirt1 and Onecut2. The enzyme Sirt1, deacetylates histones and transcription factors in an NAD-dependent manner and is important in the regulation of insulin secretion.^34^ Onecut2, a transcription factor, regulates the expression of granuphilin which negatively modifies the exocytosis of insulin-containing granules.^35^

In MIN6 and INS-1 cell lines, miR-124 regulates potassium channel subunits, Kir6.2 and SUR1, by targeting FoxA2 and leads to altering the Ca^2+^-sensitivity of β-cell and insulin secretion.^27^ Although Rab27A is a direct target of mir-124a, some other exocytosis- related proteins such as SNAP25, Rab3A, Synapsin-1A, and Noc2 are regulated by mir-124a indirectly.^36^ Recent study revealed that miRNA-463-3p/ABCG4 (ATP-binding cassette sub-family G member 4) axis plays an important role in GSIS and inhibits this process. In type 2 diabetic patients, miRNA-463-3p is up-regulated and ABCG4 is down-regulated in pancreatic β-cells.^37^

over-expression of miR-96 diminishes the exocytosis through increase in granuphilin expression and decrease in Noc2 level.^36^ Granuphilin negatively modifies exocytosis^36^ whereas Noc2 binds to Rab3 and ameliorates insulin secretion.^38^ Over-expression of miR-21 and miR-34a decreases insulin secretion by targeting VAMP2 and Rab3a.^39,40^ Additionally, miR-29a targets Syntaxin-1A and impairs the insulin secretion in glucose-dependent manner.^41^ Accordingly, miRNAs can regulate GSIS and contribute to the hyperglycemia seen in diabetes (Table 1).

Prolonged glucose stimulation activates β-cell specific insulin transcription factors such as MafA, PDX1, NeuroD and particularly miRNAs.^42^ In the hyperglycemic diabetic mouse model B6 ob/ob, augmented expression of miR-204, reduced insulin synthesis by targeting and down-regulating MafA.^43^ Over-expression of miR-9 *in vivo* decreased insulin expression via targeting Onecut2 transcription factor.^35^ MiR-30d has glucose-dependent expression and its up-regulation increases insulin transcription through indirect targeting of MafA.^44,45^ Further, continuous glucose exposure in INS-1E cells down-regulates the level of mir-375. MiR-375 directly targets PDK1 and affects the phosphorylation of PKB and GSK3, downstream kinases in the PI3-kinase signaling cascade, subsequently, inhibits glucose-induced β-cell proliferation and insulin transcription^46^ (Table 1).

**Table 1:** miRNAs acting on GSIS and insulin transcription

**miRNAs**	**functional effect**	**Targets**	**Tissue/cell**	**References**
miR-30d	Insulin transcription	MafA	MIN6	^44,45^
miR-204	Insulin transcription	MafA	mouse ob/ob islets	^22,43^
miR-375	Insulin transcription, insulin secretion	PDK1, myotrophin	Mouse islets/INS-1E	^22^
miR-9	Insulin secretion	Onecut2, Granuphilin	INS-1E	^35^
miR-21	Insulin secretion	VAMP2, Rab3a	MIN6	^39^
miR-29a	Insulin secretion	Syntaxin-1A	INS-1E	^41,98^
miR-34a	Insulin secretion	VAMP2, Rab3a	MIN6	^39^
miR-96	Insulin secretion	Noc2, Granuphilin	MIN6	^36^
miR-124a	Insulin secretion	Foxa2,Rab27a, Noc2, SNAP25	MIN6 and INS-1 832/13	^27,36^
miR -463	Insulin secretion	ABCG4	Human	^37^

The β-cells in the developing pancreas are extremely proliferative. By producing insulin via the progenitor cells, the proliferation depletes profoundly.^47^ In human, proliferation of adult pancreatic β-cells is low to undeterminable under steady-state conditions.^48,49^

Significantly, genetic studies reveal that miR-375 is one of the rare miRNAs, which its knockdown is implicated in defects in islet architecture and function of insulin producing cells.^50^ Genetic inactivation of miR-375 in zebrafish causes decrease in beta cell mass and consequently depletes insulin production and triggers the onset of diabetes.^51^ Studies on a miR-375 KO mouse model displayed genetic inactivation of miR-375 reduced β-cell mass but increased α-cell number, improved hyperglycemic state and GSIS.^52^ Indeed, appropriate level of miR-375 is also critical for expanding fetal β-cell mass and preventing abnormal glucose homeostasis.

In human, miR-7 is highly expressed in both the developing and adult pancreas.^53-55^ Transfecting human islets beta cells with anti-mir-7a demonstrated a 30-fold increase in proliferation, so it underscores the potential of miR-7 as a negative regulator of proliferation.^56^

β-cell mass in the adult human increases in response to insulin resistance (IR) during obesity and pregnancy.^48,57^ The loss of β-cell mass is associated with both type 1 (T1D) and type 2 diabetes (T2D). The hunt for finding novel mechanisms to propel β-cell to develop and regenerate can hold promise in increasing the number of functional β-cells in patients with diabetes.

Neurog3, a key regulator of β-cell differentiation, is not expressed during the regenerative phase.^28^ Profiling 283 miRNA expression levels of developing and regenerating pancreas showed that miRNAs targeting Neurog3 (miR-15a, miR-15b, miR-16 and miR-195) have the most expression during pancreas regeneration. It is plausible that, microRNAs regulate Neurog3 expression during regeneration in the adult mouse pancreas.

MiR-200 family is the principal regulator of β-cell apoptosis in T2D. In other words, miR-200 family is over-expressed in islets of diabetic mice and induces β-cell apoptosis and T2D development through targeting essential β-cell chaperone Dnajc3 (p58IPK) and the caspase inhibitor Xiap. The loss of miR-200 function protects β-cells against both oxidative and DNA damage stress and represses expression of pro-apoptotic genes.^58^ During the development of diabetes, up-regulation of miR-21 in β-cells induces apoptosis by degradation of BCL2 mRNA and inhibition of BCL2 mRNA translation^59^ (Table 2).

**Table 2:** miRNAs regulating β-cell development

**miRNAs**	**functional effect**	**Targets**	**Tissue/cell**	**References**
mir-15a, miR-15b, miR-16, miR-195	Pancreas development, β-cells fate and regeneration	Neurog3	mouse embryo/MIN6	^28^
miR-7a	Human β -cell proliferation	p70S6 K, elF4E, Mapkap1, Mknk1 and Mknk2	mouse islets	^53,56,99^
miR-375	α- and β-cell expansion	Cav1, Id3, Smarca2, Aifm1, Rasd1, Rgs16, Eef1e1, C1qbp, HuD, Cadm1	KO mouse islets	^52^
miR-124a	Pancreas development and β-cells functional	Foxa2, Neurog3	MIN6 , mouse islets	^27,28^

## MicroRNAs acting on skeletal muscle insulin sensitivity

Muscle tissue is the largest consumer of glucose in the human body. Impaired insulin-stimulated glucose uptake and glucose utilization are the characteristics of insulin resistance in skeletal muscles.^60^ Also, insulin resistance and T2D can be attributed to diminished mitochondrial function in skeletal muscle.^61^

In striated muscle, some miRNAs such as miR-1, miR-133a/b-3p, miR-206-3p, miR-208a/b-3p and miR-499-5p are the tissue-specific miRNAs with expression levels of at least 20-fold higher in comparison to other tissues.^62-64^ Additionally, theses miRNAs accounts for 25% of total miRNAs express in skeletal muscle and are termed “myomiRs”. Most myomiR family members are expressed in both cardiac and skeletal muscle with the exception of miR-208a-3p which is cardiac-specific and miR-206, which is skeletal muscle-specific. MiR-486 has expression in other tissues and considered “muscle-enriched” rather than “muscle-specific”.^63^

“mitomiR” is a term given to the miRNAs identified inside mitochondria. Of relevant importance is that miRNAs are implicated in regulation of mitochondrial biogenesis, energy metabolism, and electron transport chain subunits.^65^

Intriguingly, epigenetics regulates gene expression in response to extracellular stimuli or pathological states. Exercise is known to have beneficial effects on T2D and IR. Previous studies reveled that exercise leads to epigenetic modifications such as DNA methylation.^66^ It has been well-characterized that response to exercise in skeletal muscle is largely mediated by miRNAs which post-transcriptionally regulate gene expression. Markedly, regarding insulin sensitivity, exercise alters skeletal muscle genes and microRNAs expressions such as miR-378^67^(Table 3). Acute exercise resulted in increased levels of miR-1, miR-107, miR-181^68^ and miR-133a^69^ and diminished miR-23 levels which it can up-regulated peroxisome proliferator-activated receptor gamma coactivator 1-alpha (PGC-1α).^70^ Furthermore, through analysis of epigenetic changes in skeletal muscle of T2D patients in response to both types of exercise revealed a metabolic reprogramming.^1^

**Table 3:** miRNAs in skeletal muscle, adipose tissue and liver contribute to diabetes

**miRNAs**	**Cell types**	**functional effect**	**References**
Myomirs: miR-1, miR-133, miR-206	Skeletal muscle	Myogenesis	^100, 101^
miR-1, miR-107, miR-133, miR-181	Skeletal muscle	-	^68,69^
miR-23a	Skeletal muscle	Associated with PGC-1a upregulation	^70^
miR-29a	Skeletal muscle	Impairs insulin-stimulated glucose uptake, IRS-1	^98, 102^
miR-206	Skeletal muscle	Fiber type transition	^103^
miR-106	Skeletal muscle	-	^104, 105^
miR-24	Skeletal muscle	-	^105^
miR-27	Adipose tissue	Adipocyte differentiation	^106^
miR-29a	Adipose tissue	Impairs insulin-stimulated glucose uptake	^98^
miR-133	Adipose tissue	Adipocyte differentiation	^78^
miR-143	Adipose tissue	Adipocyte differentiation, insulin resistance	^79,107^
miR-93	Adipose tissue	Insulin resistance	^77^
miR-126	Adipose tissue	-	^105^
miR-221	Adipose tissue	Insulin resistance	^80^
miR-320	Adipose tissue	Insulin resistance	^75^
miR-193b, miR 365, miR-196a, miR-155, miR-133a/b, miR 455 and miR-30	Adipose tissue	Browning of white fat	^83^
miR-103	Adipose tissue/liver	Insulin resistance	^78^
miR-107	Adipose tissue/liver	Insulin resistance	^78^
miR-33	Liver	Controls HDL biogenesis	^87,88^
miR-122	Liver	Controls VLDL secretion	^85,108^
miR-181a	Liver	Improves insulin resistance	^90^
miR-802	Liver	Insulin resistance	^109^

## MicroRNAs acting on adipose tissue insulin sensitivity

Adipose tissue is a highly active metabolic endocrine organ and one of the important targets of insulin action.^71^ Excess adipose tissue contributes to obesity related metabolic diseases. Multiple lines of evidence underscore the importance of miRNAs in adipogenesis and obesity.^72^ Over-expressed miR-223 in adipose tissues of IR patients reduced GLUT4 protein content and subsequently impaired glucose uptake in these tissues.^73^ MiR-26b increases insulin sensitivity via the PTEN/PI3K/AKT pathway. Decreased miR-26b expression is involved in obesity-related insulin resistance in adipocytes.^74^ In insulin resistant 3T3-L1 adipocytes, miR-320 and miR-29 mediate insulin response through the PI3K/AKT pathway.^75^ Some other microRNAs such as miR-21,^76^ miR-93,^77^ miR-103, miR-107,^78^ miR-143^79^ and miR-221^80^ are concerned in insulin sensitivity of adipocytes (Table 3).

Notably, miRNAs can be packaged into exosomes and secreted from cells. Ying et al. demonstrated that miR-155 is present in obese adipose tissue macrophages exosomes and targets PPARγ. These miRNA-containing exosomes can modulate insulin sensitivity, and glucose homeostasis in insulin target tissues.^81^

Remarkably, brown adipocytes can emerge among white adipose tissue (WAT); this phenomenon is known as browning of white fat. Owing to the fact that, brown adipose tissue (BAT) increases energy expenditure rather than storage of fat, the presence of BAT in adults, hold the promise for treatment of metabolic disorders such as T2D and obesity.^82^ It has been well documented that several miRNAs such as miR-193b/-365, miR-196a, miR-155, miR-133a/b and miR-30 are instrumental in recruiting brown adipocytes in white adipose tissue.^83^

## MicroRNAs acting on liver insulin sensitivity

Hepatic insulin resistance disturbs glucose and lipid metabolism and also is a contributing factor in the pathogenesis of T2D and metabolic syndrome. MiR-122 is abundantly expressed in liver and constitutes up to 70% of all liver microRNAs. Inhibition of this microRNA is implicated in decreased hepatic steatosis, plasma cholesterol^84^ and also results in decreased circulatory lipoprotein levels through reduction of very low density lipoprotein (VLDL) secretion.^85^ Moreover, miR-223 controls cholesterol biosynthesis and high density lipoprotein (HDL) uptake in the liver.^86^ MiR-33b and miR-33a via SREBF1 and 2, respectively^87,88^ and miR-29 through regulation of Ahr and Sirt1 impact on cholesterol and lipoprotein metabolism.^89^ Additionally, MiR-181a improves hepatocyte insulin sensitivity via down-regulation of Sirt1.^90^

Liver insulin resistance results in decreased miR-338-3p expression. Several other miRNAs such az miR-143, miR-181a, miR-103, miR-107, miR-802 has been shown to improve insulin sensitivity^91^ (Table 3).

## MicroRNAs as circulating biomarkers

Large set of miRNAs besides their intracellular function are found in bio fluids, such as blood, urine and saliva.^91^ Variations in the miRNA patterns of bio fluids are emerging as promising biomarkers of several pathological conditions including diabetes.^92-96^

One of the first studies to evaluate the circulating miRNAs profile changes associated with T2DM identified most significantly changed miRNA: miR-15a, miR-126, miR-223, miR-320, and miR-28-3p were able to distinguish T2DM patients from healthy controls.^97^ Notably, the miRNAs signature sometimes is able to predict diabetes development in 70% of patients in a 10 year follow-up.

## Conclusion

Exhaustive lists of miRNAs have been implicated in the metabolic syndrome and diabetes mellitus. Although the full repertoire of miRNAs involved in β-cell differentiation and functions remains to be elucidated, more defined number of microRNAs appear to affect the function or differentiation of the pancreatic β-cells. However, microRNAs in skeletal muscle, liver and adipose tissue constitute different and almost non-overlapping sets of microRNA. Collectively, the hunt for new regulatory miRNAs in different cell types is still open.

## Acknowledgments

The authors would like to thank Dr. Louise Torp Dalgaard, Roskilde University, for the valuable comments and advice during the preparation of this paper.

## Ethical Issues

Not applicable.

## Conflict of Interest

Authors declare no conflict of interest in this study.

## Abbreviations

ABCG4 ATP-binding cassette sub-family G member 4

Ago Argonaute

BAT Brown adipose tissue

GSIS Glucose stimulated insulin secretion

HDL high density lipoprotein

IR Insulin resistance

miRNA microRNA

PGC-1α peroxisome proliferator-activated receptor gamma coactivator 1-alpha

RIP Rat insulin promoter

RISC RNA-induced silencing complex

SNARE Soluble NSF-attachment protein receptor

T1D Type 1 diabetes

T2D Type 2 diabetes

UTR Untranslated region

VLDL very low density lipoprotein

WAT White adipose tissue
